# Study of Enzymatically Treated Alginate/Chitosan Hydrosols in Sponges Formation Process

**DOI:** 10.3390/polym8010008

**Published:** 2016-01-05

**Authors:** Anna Zimoch-Korzycka, Dominika Kulig, Andrzej Jarmoluk, Krzysztof Marycz, Weronika Matuszczak

**Affiliations:** 1 Department of Animal Products Technology and Quality Management, Faculty of Food Science, Wrocław University of Environmental and Life Sciences; 37 Chelmonskiego St.; 51-630 Wrocław, Poland; dom.kulig@gmail.com (D.K.); andrzj.jarmoluk@up.wroc.pl (A.J.); matuszczak.w@gmail.com (W.M.); 2 Department of Environment Hygiene and Animal Welfare, The Faculty of Biology and Animal Science, Wrocław University of Environmental and Life Sciences; 38 C Chelmonskiego St.; 50-630 Wrocław, Poland; krzysztof.marycz@up.wroc.pl

**Keywords:** hydrosol, sponge, sodium alginate, chitosan, lysozyme

## Abstract

The aim of the study was to produce 3D sponges based on enzymatically modified lysozyme selected polysaccharides and assess their physicochemical properties. The alginate/chitosan sponges were formed from polymers hydrosols in different proportions at a final concentration of 1% polysaccharides. Hydrosols were modified by lysozyme addition of 1000 U. Hydrosols without or with enzyme were analyzed for their reducing sugar content, rheological properties and ability to scavenge free radicals. Sponges formed from hydrosols were tested for solubility and compressive properties. Only chitosan was hydrolyzed by lysozyme. The morphology of sponges was investigated by scanning electron microscopy (SEM). It was proven that the antioxidant properties of hydrosols are dependent on the concentration of chitosan. It was also shown that the addition of lysozyme negatively affected the free radical scavenging ability of single hydrosols of alginate and chitosan, and their mixtures. The Ostwald de Waele as well as Herschel–Bulkley models of rheological properties fitted the experimental data well (*R*^2^ is between 0.947 and 1.000). Increase in textural features values of sponges was observed. Sponges with pure alginate and pure chitosan were almost completely soluble. The enzyme addition significantly changed the characteristics of the cross-section structure of sponges, and made the surface smoother.

## 1. Introduction

The dynamic development of tissue engineering and search for new biocompatible substances over the last two decades has changed the proportions of used biomaterials. While at the end of the 1950s, the most popular were metallic materials and their alloys, in the following years interest in polymers increased. Polymers can be widely used in biomedical and food applications. They are biodegradable, biocompatible, non-toxic, reactive, bioactive and have water-binding capacity. In nature, it is hard to find a perfect polymer, which would possess all of these features. New materials that combine optimal mechanical, physical, chemical and biological features are being looked for currently. The expectations of these properties are quite broad and include mechanical properties as elastic modulus, shear modulus, yield strength, tensile strength and resistance to cracking and fatigue. Important parameters of physical conditions are: density, topography, roughness, shape, or electric and magnetic properties. Chemical structure, adhesion, biofunctionality, bioactivity, and biodegradability are the advantages of chemical and biological agents. During manufacture or treatment of such material, the essential features are reproducibility, ease of sterilization and reliability. The solution appears to be composites consisting of at least two polymers with different properties. Based on ionic charges, polymers can be classified as neutral (no charge), anionic (negatively charged), cationic (positively charged) and ampholytic (positively and/or negatively charged). Natural anionic polymers include hyaluronic acid, sodium alginate, pectin, carrageenan, chondroitin sulfate, and dextran sulfate, whereas cationic polymers include chitin, chitosan and polylisin. Natural amphipathic polymers include collagen, carboxymethyl chitin, fibrin and dextran, whereas agarose and pullulan are examples of neutral polymers [1]. More and more frequently, chitosan is used in this type of application, in view of its rich biochemical importance. Biodegradability, biocompatibility, non-toxicity, hemostatic, antibacterial, regenerative and antioxidant properties of chitosan have already been reported [2,3,4] as well as ability to form 3D structures for tissue engineering [5]. In addition, sodium alginate is a natural, biodegradable, inexpensive polymer, whose hydrogel is structurally similar to extracellular living tissue and is an excellent carrier of biologically active substances allowing its use in medical, pharmaceutical and food applications [6,7]. Unfortunately, pure alginate scaffold is unable to support cell metabolism, because low bioactive properties cannot interact in the molecular structures [8,9,10]. Sodium alginate and chitosan are oppositely charged polyions, which form polycation–polyanion (polyelectrolyte) complexes as a result of an electrostatic mechanism [11]. Creation of a film with naturally formed porosity and placement of –COO– groups for binding to Fe(II) ions is possible in appropriate ratio of alginate/chitosan complex [12]. Highly porous material with large surface to volume ratio is important for nutrient supply of cells and enhances their diffusion rate [13]. Simultaneously, alginate and chitosan are susceptible to various types of degradation, enzymatic, acid, alkaline, and oxidation-reduction, which results in cleavage of glycosidic linkages and final depolymerization. The degree of degradation depends primarily on the concentration of reactants and reaction temperature [14]. Enzymatic modification of individual chitosan by lysozyme, papaine, α-amylase, and chitosanase is already known [15,16,17]. Hydrolysis of alginate, by alginate lyase, was also reported [18], but simultaneous effect of alginate/chitosan hydrosols and sponges with lysozyme on antioxidant, rheological, compressive, morphology or solubility properties is novel.

The aim of the study was to produce 3D sponges based on selected polysaccharides (sodium alginate, chitosan) enzymatically modified by lysozyme and assess their physicochemical properties.

## 2. Materials and Methods

### 2.1. Materials

Low molecular weight chitosan (DD = 75%–85%; *M*_w_ = 150 kDa) and dl lactic acid (85% syrup) were obtained from Sigma Aldrich, Poznan, Poland. Alginate FD 901 AR was purchased from Danisco GRINDSTED^®^, Grindsted, Denmark. Lysozyme from white egg hen with 2000 U/mg activity was obtained from Ovopol, Nowa Sol, Poland.

### 2.2. Preparation of Enzyme/Alginate/Chitosan Hydrosols and Sponges

Chitosan (C) hydrosol was prepared in 1% aqueous solution of lactic acid by stirring at 400 rpm for 20 h. Alginate sodium (A) hydrosol was prepared in an analogous way. Enzyme: lysozyme (L) was dissolved in water by stirring at 300 rpm for 30 min and was added to A in activity of 1000 U. Then, both polymers (1%) were homogenized by homogenizer IKA (T18 basic, Ultra Turrax, Staufen, Germany) for 90 s in proportions shown in Table 1. The mixtures were poured into polypropylene beakers of 60 mL and frozen at −80 °C for 24 h. Afterwards, the mixtures were lyophilized at −56 °C for 7 days.

**Table 1:** Enzyme/alginate/chitosan hydrosols composition.

Enzyme Addition (E)	Polymers Ratio A:C	Variants (E/A/C)
(U)	(*v*/*v*)	(code)
N *	0:1	N/C
	1:3	N/A/3C
	1:1	N/A/C
	3:1	N/3A/C
	1:0	N/A
1000 L	0:1	L/C
	1:3	L/A/3C
	1:1	L/A/C
	3:1	L/3A/C
	1:0	L/A

* no enzyme addition; A, sodium Alginate; C, Chitosan.

### 2.3. Reducing Sugar Assay of Enzyme/Alginate/Chitosan Hydrosols

The reducing sugar was determined using 3,5-dinitrosalicylic acid (DNS) as reagent and calculated as glucose. DNS reagent solution contains 3,5-dinitrosalicylic acid (1 g), 2 M NaOH (20 mL) and potassium sodium tartrate (30 g) dissolved in distilled water (100 mL) according to Miller [19]. The same volumes of samples and DNS reagent were mixed and then boiled for 8 min. After cooling, the absorbance of the clear solution was measured at 540 nm using an UV 2601 RAYLEIGH spectrometer.

### 2.4. Antioxidant Activity of Enzyme/Alginate/Chitosan Hydrosols

Free radical scavenging activity of the hydrosols was determined by the method of Yamaguchi *et al.* [20]. Hydrosols were 20 times dissolved in water and 1.0 mL was taken and mixed with 1.0 mL of 0.1 mM 2,2-diphenyl-1-picrylhydrazyl (DPPH)–methanol solution. The reaction mixture was shaken well and incubated for 30 min at ambient temperature. The reduction of the DPPH free radical was measured by reading the absorbance at 517 nm. Control sample was DPPH–methanol solution. The antioxidant activity was calculated from the standard curve and expressed in μM Trolox/mL needed to neutralize 0.15 mM solution of DPPH free radicals.

### 2.5. Rheological Characterization of Enzyme/Alginate/Chitosan Hydrosols

Rheological characterization of the hydrosols was performed in a Haake RS 6000 rotational viscometer (Haake, Karlsruhe, Germany) following the methodology described by Garcia *et al.* [21]. The measurement was made at a constant temperature (20 °C) using a system of coaxial cylinders with conical rotor Z20 DIN. The sample (8.2 mL) was applied to the cylinder. The sample was stabilized for 3 min at 20 °C. The shear stress was determined as a function of shear rate in the range of 0–512 s^−1^, following the program:

- 3 min to a maximum shear rate;
- 1 min at maximum shear rate; and
- 3 min to reach a shear rate of 0.

Flow curves were plotted following the Herschel–Bulkley and the Ostwald de Waele models. Apparent viscosity was determined at a shear rate of 256 s^−1^ [21].

The power model of Ostwald de Waele is the simplest, two-parameter rheological model that describes the flow curves of the test medium [22].

(1) $$\tau =k\cdot \gamma^{n}$$

or

(2) $$\eta_{\alpha }=k\cdot \gamma^{n-1}$$

where:

𝜏—shear stress (Pa);
k—consistency index (Pa·s);
γ—shear rate (s^−1^);
*n*—flow behavior index (–);
η_α_—apparent viscosity (mPa·s).

The Herschel–Bulkley’s model is the simplest model of flow curves of nonlinear viscoelastic liquids. The parameters of this model are: 𝜏_0_, *k*, *n* [23].

(3) $$\tau =\tau_{0}+k\cdot \gamma^{n}$$

where: 𝜏_0_—yield stress (Pa)

### 2.6. Compressive Properties of Enzyme/Alginate/Chitosan Sponges

The compressive properties of sponges were measured by texturometer Zwick/Roell Z010. The test was conducted in accordance with ASTM D1621 [23]. The speed of the head was 200 mm/min, the initial force of 0.17 Pa, and the maximum deformation of the sample was 20%. All sponges were tested in triplicate. Compressive strength and modulus of elasticity were recorded. Compressive strength (σ^10^) is the stress at the yield point if a yield point occurs before 10% deformation or, in the absence of such a yield point, the stress at 10% deformation (kPa). Modulus of elasticity (*E*) is the ratio of stress (nominal) to corresponding strain below the proportional limit of a material expressed in force per unit area based on the minimum initial cross-sectional area (kPa).

### 2.7. Solubility of Enzyme/Alginate/Chitosan Sponges

Solubility test was performed as described by Amid and Mirhosseini [24]. Sponges were stored in a desiccator (0% RH) for 7 days and weighed to the nearest 0.0001 g and placed into test beakers with 100 mL deionized water. The samples were maintained under constant agitation for 24 h at room temperature (approximately 25 °C). The remained pieces of sponges, after soaking through the filters, were dried at 60 °C for 30 min to constant weight. The percentage of total soluble matter (% solubility) was calculated as follows:

(4) %Solubility = Final dry weight/Initial dry weight × 100

### 2.8. Scanning Electron Microscopy of Enzyme/Alginate/Chitosan Sponges

Microstructure of surface and cross-section of 3D sponges were evaluated using EVO LS15 ZEISS Scanning Electron Microscopy (Zeiss, Jena, Germany). The sponges were cut out into samples with surface 0.5 mm^2^ and sputtered with gold for 150 s using a sputter coater Scancoat 6 (Edwards, London, UK), which finally generated a 10 nm thick gold layer. Each coated sample was examined using a voltage of 20 kV.

### 2.9. Statistical Analysis

All experiments were triplicated. The effects of two independent categorical variables, such as proportion of C and A and no addition or addition of L, were evaluated. Due to the mutual entanglement of the two variables, directly dependent on each other, *i.e.*, chitosan and alginate, the effect of interactivity was considered depending on the ratio of the two polymers and the addition of the enzyme. Data were analyzed by two-way factor analysis of variance (ANOVA) with a significance level defined at *p* ≤ 0.05 using Statistica 9. Data of rheological properties of hydrosols and pore size of sponges were analyzed by one-way factor analysis of variance (StatSoft, Krakow, Poland) [25].

## 3. Results and Discussion

### 3.1. Reducing Sugar

**Figure 1:**
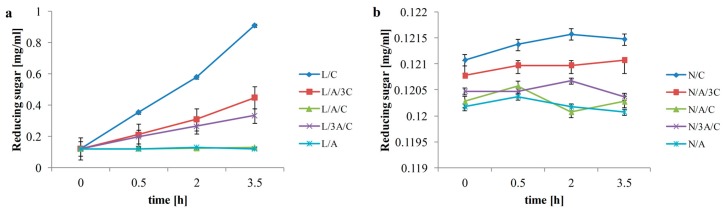
Reducing sugar content in hydrosols with lysozyme (**a**) and without (**b**).

The enzyme activity against alginate, chitosan and their mixture is showed as reducing sugar content in Figure 1a. The lysozyme action is clearly seen on chitosan, but there is no hydrolysis effect on alginate. Alginic acid or alginate consists of mannuronic acid and guluronic acid linked through 1–4 linkage that cannot be identified by lysozyme. Therefore the alginate is stable in presence of this enzyme. The lysozyme only recognizes glycosidic linkages between *N*-acetylglucosamine units [26]. Alginate can be hydrolyzed by alginate lyases. Double bond between C4 and C5 is formed as the result of catalyzation of the β-elimination of the 4-*O*-linked glycosidic bond. The products are unsaturated uronic acid-containing oligosaccharides [27]. An equal volume of polymers caused stopping enzymatic reaction. The amount of reducing sugar of hydrosols without lysozyme addition is presented in Figure 1b.

### 3.2. Antioxidant Activity

The results of statistical analysis of the antioxidant properties of alginate and chitosan hydrosols, and their mixtures with or without the enzyme: lysozyme and their interactions are shown in Table 2. The use of enzyme as well as polymer ratio was statistically significant on scavenging ability to DPPH radicals of hydrosols. The highest antioxidant property was noticed for polymer ratio 1:1 and equalled 205.11 μM Trolox. The addition of lysozyme was significant for the antioxidant properties and caused reduction of this parameter value to 132.43 μM Trolox, whereas hydrosols composed without enzyme exhibit stronger scavenging ability—198.41 μM Trolox.

Interaction effects of polymer ratio and use of enzyme on free radical scavenging were noted for all hydrosols. The highest and the lowest values of 231.33 μM Trolox and 80.00 μM Trolox were observed for N/C and L/A, respectively. The more chitosan in hydrosols composition, the better the antioxidant effect. Chitosan possesses strong antioxidant properties, which was confirmed by Muzzarelli *et al.* [28] who noted that this polymer and its derivatives inactivate superoxide and hydroxyl radicals. The antioxidant properties are dependent on the concentration of the active ingredient. Chitosan in a higher concentration is a much stronger scavenger [28]. Hydrosols with lysozyme had the lowest free radical scavenging activity compared to hydrosols without enzyme. The ability of chitosan, hydroxypropyl methylcellulose and nano silver hydrosols to scavenge free radicals increases with lysozyme addition [28]. The antioxidant properties of hydrosols of chitosan and lysozyme are weaker than in composition presented by Zimoch-Korzycka and Jarmoluk [29].

**Table 2:** Antioxidant activity of hydrosols (main effects and interactions).

Main effects				Interactions A/C	
A/C		E			
Polymers ratio (*v*/*v*)	Free radical scavenging of DPPH (μM·trolox/mL)	Polymers ratio (*v*/*v*)	Free radical scavenging of DPPH (μM·trolox/mL)	Polymers ratio (*v*/*v*)	Free radical scavenging of DPPH (μM·trolox/mL)
0/1	202.89 ± 21.15 ^b^	N	198.41 ± 10.48 ^a^	N/C	231.33 ± 10.67 ^g^
				N/A/3C	208.43 ± 2.02 ^def^
1/3	181.71 ± 15.19 ^a^			N/A/C	220.27 ± 5.60 ^fg^
				N/3A/C	206.01 ± 4.19 ^def^
1/1	205.11 ± 4.39 ^b^			N/A	126 ± 13.15 ^b^
		L	132.43 ± 10.08 ^b^	L/C	120.66 ± 3.13 ^b^
3/1	186.71 ± 10.13 ^a^			L/A/3C	121.33 ± 1.53 ^b^
				L/A/C	193.33 ± 2.98 ^d^
1/0	119.11 ± 11.32 ^c^			L/3A/C	146.8 ± 4.05 ^c^
				L/A	80 ± 7.55 ^a^

^a–h^ values with different letters within the same column differ significantly (*p* < 0.05); A, sodium Alginate; C, Chitosan; E, enzyme; N, no enzyme addition ; L, Lysozyme

### 3.3. Rheological Properties of Hydrosols

**Figure 2:**
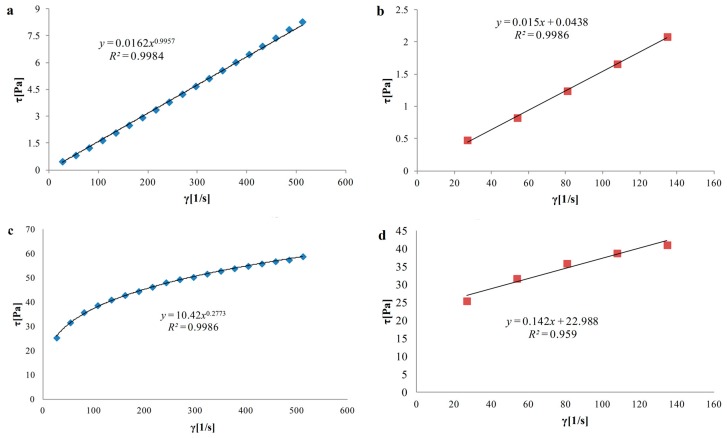
Flow curve (**a**) and yield stress determination (**b**) of N/C hydrosol and flow curve (**c**) and yield stress determination (**d**) of L/A/C hydrosol.

The rheological parameters obtained for all the variants using the Ostwald de Waele and the Herschel–Bulkley models are shown in Table 3. The Ostwald de Waele model as well as Herschel–Bulkley model fitted the experimental data well. All *R*^2^ values are larger than 0.9 (0.947–1.000). Hydrosols consisted of alginate/chitosan blend showed hysteresis. The size of the hysteresis was related with polymer ratio. All experimental samples showed pseudoplastic behavior (*n* ˂ 1). It means that non-Newtonian behavior was observed in every sample (*n* ≠ 1). Flow curve and determination of the yield stress of N/C and L/A/C hydrosols are presented in Figure 2a–d, respectively. Synergistic, positive effect of polymers ratio and enzyme addition is seen in apparent viscosity (*η*) of L/A/C and N/A/C hydrosols, which are much higher than other variants. Meng *et al.* [11] mixed chitosan and alginate in various proportions and obtained membranes tested for viscosity. They found out that chitosan/alginate membrane in ratio 1:1 presented the highest viscosity and hypothesized that it may be related to the changes of electrostatic potential during naturalization inducing polyelectrolyte complexation. It was confirmed by FTIR and XRD that chitosan/alginate polyion complex was formed in ratio 1:1 [11]. When the fluid becomes more viscous, *k* increases, and when the fluid becomes shear-thinned then *n* decreases [30]. The flow index is generally not sensitive on temperature changes [31]. Different solutions have different characteristics of flow curves, which are dependent on the variables of the relationship between viscosity and the shear rate of mixed solutions [32]. The consistency index (*k*) gives an idea of the solution viscosity. However, *n* values have to be considered to compare *k* values of various solutions [33]. The *k* showed very similar values for the variants of the same polymer ratio and differentiated by enzyme use. For example, *k* of N/C and L/C hydrosols were equal to 0.02 and 0.01 Pa·s respectively and this difference was not statistically significant. Hydrosols with low concentration of polymer can have yield stress close to zero [34]. The yield stress is not observed in polymer solutions where particles sediment very quickly [35]. As the alginate content increased, the yield stress decreased, with the exception of samples with stoichiometric concentrations of polymers and enzyme use (L/A/C = 22.99 Pa). The high yield stress of this hydrosol is an advantageous feature. It provides good spreading and adhesion to materials. The addition of magnesium aluminum silicate to chitosan dispersions provides high viscosity and a change in flow type of chitosan from Newtonian to pseudo-plastic flow with thixotropic properties [36]. Authors explained this change in the behavior by electrostatic interaction between a positive and negative charge of chitosan and magnesium aluminum silicate, respectively. Following this findings, it was assumed that the oppositely charged alginate and chitosan modified by enzyme in hydrosols can be also applied as suspending and gelling agents in food and pharmaceutical products.

**Table 3:** Rheological properties of hydrosols.

Variants (E/A/C)	Ostwald de Waele model			Herschel–Bulkley model
	Consistency index *k* (Pa·s)	Flow behavior index *n* (–)	Apparent viscosity *η* (mPa·s)	Yield stress *τ_0_* (Pa)
N				
N/C	0.02 ± 0.01 × 10^−3 a^	0.996 ± 0.05 × 10^−2 c^	15.6 ± 0.1 ^a^	0.04 ± 0.02 × 10^−3 a^
N/A/3C	0.17 ± 0.01 × 10^−2 b^	0.682 ± 0.03 × 10^−2 b^	30.5 ± 0.5 ^b^	0.94 ± 0.05 × 10^−2 b^
N/A/C	0.04 ± 0.50 × 10^−2 d^	0.901 ± 0.04 × 10^−2 g^	317.4 ± 0.4 ^g^	2.22 ± 0.1 × 10^−2 d^
N/3A/C	2.46 ± 0.10 × 10^−2 e^	0.492 ± 0.2 × 10^−2 a^	151 ± 1.0 ^e^	9.62 ± 0.50 × 10^−2 h^
N/A	0.68 ± 0.03 × 10^−2 c^	0.713 ± 0.03 × 10^−2 f^	140 ± 0.0 ^c^	2.86 ± 0.10 × 10^−2 f^
L				
L/C	0.01 ± 0.49 × 10^−5 a^	0.997 ± 0.05 × 10^−2 c^	14.4 ± 0.1 ^a^	0.01 ± 0.49 × 10^−5 a^
L/A/3C	0.18 ± 0.09 × 10^−3 b^	0.677 ± 0.03 × 10^−2 b^	28.6 ± 0.4 ^b^	1.04 ± 0.05 × 10^−2 c^
L/A/C	10.42 ± 0.51 × 10^−2 g^	0.277 ± 0.01 × 10^−2 d^	189.8 ± 0.2 ^f^	22.99 ± 0.01 ^i^
L/3A/C	2.50 ± 0.12 × 10^−2 f^	0.495 ± 0.02 × 10^−2 a^	139.5 ± 0.5 ^c^	9.16 ± 0.45 × 10^−2 g^
L/A	0.69 ± 0.03 × 10^−2 c^	0.710 ± 0.04 × 10^−2 e^	129.8 ± 0.2 ^cd^	2.38 ± 0.12 × 10^−2 e^

^a–i^ values with different letters within the same column differ significantly (*p* < 0.05).

### 3.4. Compressive Properties of Sponges

The results of statistical analysis of compressive strength and elasticity modulus of 3D sponges are shown in Table 4. The significant effect of the alginate/chitosan ratio on compressive strength value was observed. The lowest compressive strength of 0.60 kPa and 0.96 kPa was noted for the sponges of pure alginate and pure chitosan respectively. The highest resistance of sponges was found for polymer ratio 3:1 and was 4.31 kPa. The addition of lysozyme influenced significantly the compressive strength of sponges causing a decrease of this parameter. The lysozyme is able to hydrolyze β (1–4) glycosidic linkage between *N*-acetylmuramic acid and *N*-acetylglucosamine in the cell wall of polysaccharides, including bacteria [26]. Hydrolysis of chitosan by lysozyme is known in literature [37,38]. Elasticity modulus depends mainly on polymer ratio. There is no significant difference in modulus values for individual chitosan and alginate sponges. It was noted that lysozyme might decrease the elasticity of modulus. The interaction between polymers ratio and the kind of enzyme was statistically significant of all variants analyzed for compressive strength. However, the increase in compressive strength was observed with lowering addition of C until polymer ratio was 3/1. This relation was also exhibited by samples with the same polymer ratio and enzyme addition (L/3A/C). High strength of this sponge could be attributed to the interaction between cationic chitosan and anionic alginate. The same hypothesis was suggested by Archana *et al.*, who compared compressive strength of chitosan-pectin, chitosan-alginate and chitosan-pectin-alginate scaffold and concluded that the ionic interaction in chitosan and pectin with sodium alginate is stronger than in chitosan-pectin scaffold [4]. Values of elasticity modulus rise with the same trend as values of compressive strength of sponges. Ikeda *et al.* [39] suggested that mechanical properties of porous materials depend on their porosity. The smaller the pores, the lower the strength and Young modulus of sponges obtained.

**Table 4:** Compressive strength and modulus elasticity of sponges (main effects and interactions).

Main effects						Interactions A/C		
A/C			E					
Polymers ratio (*v*/*v*)	σ^10^ (kPa)	E (kPa)	Enzyme addition	σ^10^ (kPa)	E (kPa)	Variants (E/A/C)	σ^10^ (kPa)	E (kPa)
0/1	0.96 ± 0.27 ^a^	8.81 ± 1.97 ^a^	N	3.02 ± 0.69 ^a^	27.38 ± 5.28 ^a^	N/C	1.48 ± 0.31 ^ab^	11.89 ± 2.96 ^a^
						N/A/3C	3.47 ± 0.35 ^b^	31.17 ± 3.32 ^c^
1/3	2.86 ± 0.33 ^b^	22.52 ± 4.32 ^b^				N/A/C	2.23 ± 0.92 ^ab^	26.14 ± 7.33 ^bc^
						N/3A/C	7.14 ± 1.76 ^c^	59.01 ± 9.81 ^d^
1/1	2.08 ± 0.44 ^ab^	26.54 ± 3.43 ^b^				N/A	0.77 ± 0.22 ^a^	8.71 ± 1.62 ^a^
			L	1.31 ± 0.23 ^b^	21.27 ± 4.52 ^b^	L/C	0.45 ± 0.10 ^a^	5.74 ± 1.11 ^a^
3/1	4.31 ± 1.50 ^c^	55.17 ± 4.78 ^c^				L/A/3C	2.27 ± 0.28 ^ab^	13.87 ± 2.79 ^ab^
						L/A/C	1.92 ± 0.34 ^ab^	26.93 ± 2.25 ^bc^
1/0	0.60 ± 0.12 ^a^	8.60 ± 0.76 ^a^				L/3A/C	1.49 ± 0.50 ^ab^	51.32 ± 1.84 ^d^
						L/A	0.44 ± 0.03 ^a^	8.49 ± 0.47 ^a^

^a–d^ values with different letters within the same column differ significantly (*p* < 0.05).

### 3.5. Solubility

**Figure 3:**
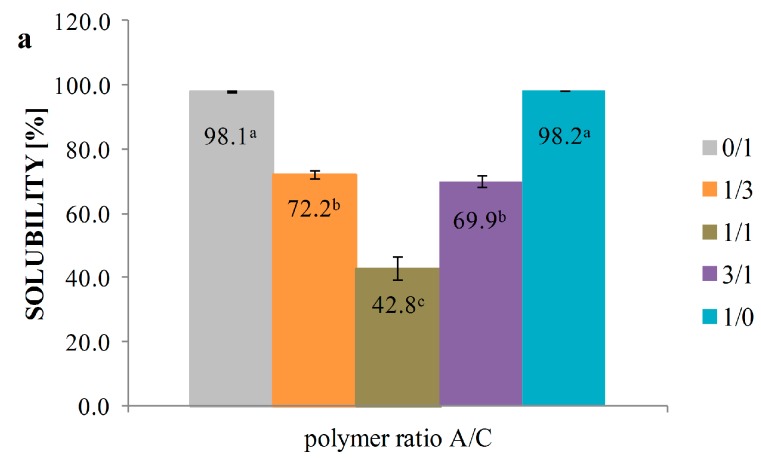

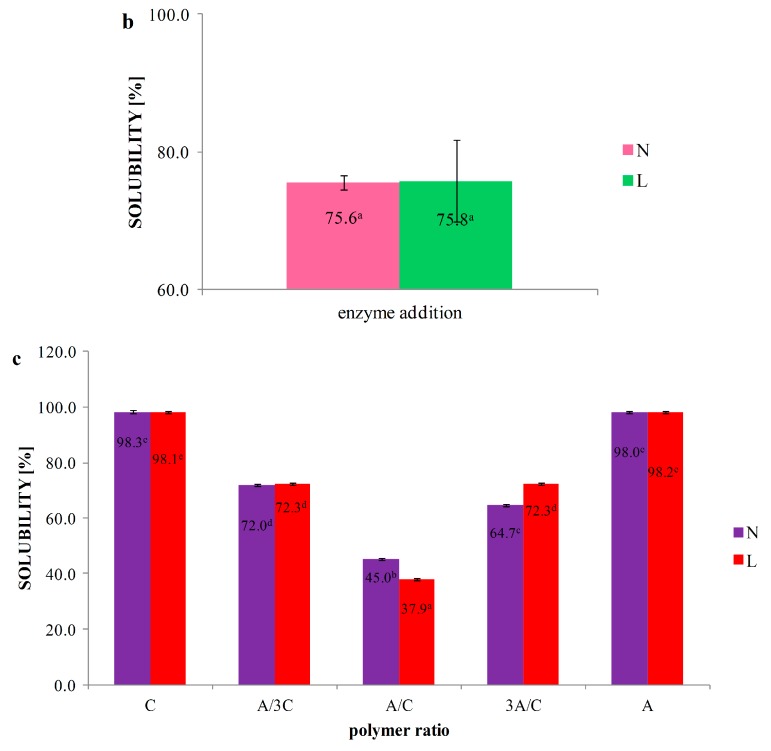
Solubility of sponges: (**a**) main effect of polymer ratio; (**b**) main effect of enzyme addition; and (**c**) interactions effect. ^a–e^ values with different letters within the same column differ significantly (*p* < 0.05; І—standard deviation).

Main effects and interactions of soluble property of 3D sponges are shown in Figure 3a–c. Equalization of the ratio between alginate and chitosan in sponge compositions reduced the solubility to 42.8%. Total concentration of alginate sponge or chitosan sponge caused almost complete dissolution. The same sponges solubility was noted before and after adding lysozyme. Ilina *et al.* [40] performed hydrolysis by enzyme preparations: β-glycanase, xylanase and cellulase of high molecular weight chitosan to obtain water-soluble chitosan. The interaction effects confirmed similar solubility of N/C, L/C, N/A, L/A hydrosols, which exhibited the highest ability of being soluble. The L/A/C sponge exhibited the lowest solubility (37.9%). An equivalent quantity of the opposite charged ions, such as chitosan and alginate, provides formation of neutrally charged polyelectrolyte complex, which is generally insoluble in water. Partially soluble in aqueous solutions are non-stoichiometric complexes, which are characterized by an excess of a specified charge (positive or negative) [41].

### 3.6. Scanning Electron Microscopy

**Figure 4:**
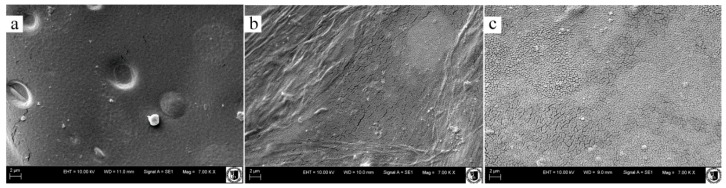
SEM images of the surfaces of N/3A/C (**a**); X/3A/C (**b**); and L/3A/C (**c**) variants.

**Figure 5:**
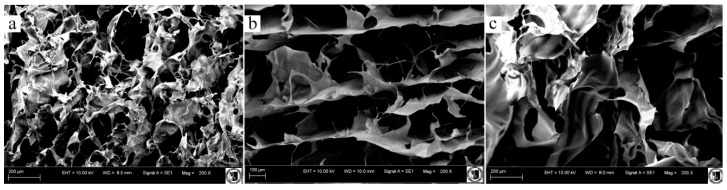
SEM images of the cross-sections of N/3A/C (**a**); X/3A/C (**b**); and L/3A/C (**c**) variants.

The surface structure of the chosen 3D sponges is presented in Figure 4a–c. The surface is smoother after adding enzyme, which could be caused by chito-oligomers. A shorter chain may stop the gap in the surface structure of sponges. The cross-section morphology is shown in Figure 5a–c. L/3A/C and N/A sponges are characterized with big irregular pores in comparison to the chitosan variant of sponge structures. The addition of enzyme caused enlargement of pore size in sponges. A similar result was observed by Gershon and Nussinovitch [42] who characterized enzymatically produced agar-starch sponges. The pore size is in correlation with mechanical properties—the larger the pore, the higher the mechanical properties, such as compressive strength and elasticity of modulus. The porous structure of the material is obtained by sublimation of the solvent in the stage of material formation and as a result of gradual resorption of the fiber component in the material. Larger pores and irregularity may result from the lack of fibrillary network formation before the freezing process. Mixed polymers can interact during freezing [43]. Besides temperature, factors determining the porous structure include: the type and the concentration of the initial reactants, pH and method of structure manufacturing and the crosslink density [1]. The pore sizes of sponges are shown in Table 5. The structure of alginate sponge exhibited bigger pores than cross-linked alginate-chitosan structures. Decreasing cross-linking agent and sodium alginate concentrations in production of hydrogels for tissue engineering causes lower density and compactness, respectively, which was shown by Wrzeszcz *et al.* [44].

**Table 5:** Pore size of cross-sectioned sponges.

Variants (E/A/C)	Pore size (μm)
N	
N/C	109.2 ± 20.2 ^a^
N/A/3C	132.8 ± 22.9 ^ab^
N/A/C	124.9 ± 15.2 ^ab^
N/3A/C	140.6 ± 21.9 ^ab^
N/A	189.8 ± 26.8 ^bc^
L	
L/C	117.6 ± 17.8 ^ab^
L/A/3C	217.6 ± 19.8 ^c^
L/A/C	120.0 ± 12.4 ^ab^
L/3A/C	218.4 ± 35.4 ^c^
L/A	224.6 ± 36.1 ^c^

^a–c^ values with different letters within the same column differ significantly (*p* < 0.05).

## 4. Conclusions

Depending on the type, enzyme use, and the interactions between polymers and the enzyme, a pseudo-plastic behavior might be observed. Varied solubility of sponges may be obtained by controlling the concentration of chitosan, which allows a wide range of applications. Synergistic influence of polymer ratio and lysozyme addition on compressive properties was clearly shown. The porosity of the 3D biocomposite sponges is in correlation with mechanical properties, which were improved by the use of enzyme. Alginate/chitosan hydrosols with or without enzymes are suitable materials for producing sponges with desired properties. Furthermore, more analysis such as infrared spectroscopy analysis and *in vitro* cell culture experiment must be done and are under investigation. We assumed that obtained sponges might be a valuable tool for *in vitro* cellular test using osteo and/or chondral progenitor cells or even neural progenitors.
